# Respiratory Syncytial Virus Infection as a Precipitant of Thyroid Storm in a Previously Undiagnosed Case of Graves' Disease in a Prepubertal Girl

**DOI:** 10.1155/2011/138903

**Published:** 2011-03-15

**Authors:** IvyR Aslan, ElizabethA Baca, RWilliam Charlton, StephenM Rosenthal

**Affiliations:** 1 Division of Endocrinology, Department of Pediatrics, University of California, San Francisco, CA 94143, USA; 2 Department of Pediatrics, Lucile Packard Children's Hospital at Stanford, Palo Alto, CA 94304, USA; 3 Division of Pediatric Endocrinology, University of California, San Francisco, 513 Parnassus Avenue, Suite S-672-D, San Francisco, CA 94143-0434, USA

## Abstract

Graves' disease is less common in prepubertal than pubertal children, and initial presentation with thyroid storm is rare. We report an 11-year-old prepubertal Hispanic girl who presented with a one-day history of respiratory distress, fever, and dysphagia. She had exophthalmos, a diffuse bilateral goiter and was agitated, tachycardic, and hypertensive. Nasal swab was positive for respiratory syncytial virus (RSV). She was diagnosed with thyroid storm and admitted to the pediatric intensive care unit. While infection is a known precipitant of thyroid storm and RSV is a common pediatric infection, to the best of our knowledge, this is the first reported case of RSV infection apparently precipitating thyroid storm in a prepubertal child.

## 1. Case Report

A prepubertal 11-year-old Hispanic girl presented to the emergency department with a one-day history of respiratory distress, fever, and dysphagia that developed after several days of upper respiratory infection symptoms and sore throat. 

Her past medical history was notable for prematurity (26 weeks gestational age) associated with chronic lung disease. On exam she was noted to be thin, agitated, drooling, in moderate-to-severe distress, and holding herself in a tripod position. Her temperature was 38.6 (C) or 101.4 (F), heart rate 200, blood pressure 162/93 mmHg, respiratory rate 40, O2 sat 91% in room air. She had diffuse erythema of the pharynx, exophthalmos and a diffuse bilateral goiter, each lobe measuring . There were no palpable nodules or tenderness, but a bruit was present. On her lung exam she had bilateral wheezes. She was prepubertal.

Laboratory Tests

Nasal swab Direct Fluorescent Antibody was positive for respiratory syncytial virus (RSV). Table 1 shows thyroid function tests at diagnosis and 1 week after treatment was started (see hospital course).

**Table 1:** Laboratory profile from the patient at diagnosis and 1 week posttreatment.

**Labs (with normal ranges)**	**At diagnosis**	**1 week posttreatment**
TSH (0.37–4.42 mIU/L)	0.01	—
Free T4 (0.65–1.80 ng/dl)	8.17	1.24
Free T3 (335– 480 pg/dl)	1918	451

The patient's chest X-ray showed linear subsegmental densities in both lungs bases as well as prominence of interstitial markings in the lung apices. Multiple cuffed bronchi are seen in the perihilar regions and in the left upper lobe. All these findings are compatible with viral pneumonia.

The following studies were also obtained at baseline: thyroid stimulating immunoglobulin 257% baseline (normal <125%), anti-thyroperoxidase antibody 4.77 IU/ml (normal <0.8), and anti-thyroglobulin antibody 68 IU/mL (normal <20). 

The patient's score of 60 met criteria for thyroid storm based on Burch and Wartofsky's criteria. A score of 45 or more is highly suggestive of thyroid storm (see Table 2) [1].

**Table 2:** Burch and Wartofsky's criteria for thyroid storm (score of 45 or more is highly suggestive of thyroid storm). The patient's scores are noted in bold.

Temperature	

99–99.9	5
100–100.9	10
101–101.9	**15**
102–102.9	20
103–103.9	25
≥104	30

CNS effects	

Absent	0
Mild (agitation)	**10**
Moderate (delirium, psychosis, extreme lethargy)	20
Severe (seizures, coma)	30

Gastrointestinal-hepatic dysfunction	

Absent	**0**
Moderate (diarrhea, N/V, abdominal pain)	10
Severe (unexplained jaundice)	20

Cardiovascular dysfunction	

90–109	5
110–119	10
120–129	15
130–139	20
≥140	**25**

Congestive heart failure	

Absent	**0**
Mild (pedal edema)	5
Moderate (bibasilar rales)	10
Severe (pulmonary edema)	15

Atrial fibrillation	

Absent	**0**
Present	10

Precipitating event	

Absent	0
Present	**10**

Hospital Course

The patient was initially treated with propylthiouracil (PTU), saturated solution of potassium iodide (SSKI), dexamethasone, esmolol infusion, and bronchodilators. She was discharged home on PTU 100 mg PO Q8h, atenolol 50 mg once daily and levalbuterol inhaled. One month after her hospital discharge, she was switched from PTU to methimazole, and the atenolol was discontinued.

## 2. Discussion

Graves' disease is less common in prepubertal than pubertal children [2], and initial presentation with thyroid storm is rare. Infection is a known precipitant of thyroid storm. While medical treatment for RSV is most common in infants, RSV infection is common in all age groups [3]. However, to the best of our knowledge there are no previous case reports of RSV infection precipitating thyroid storm in prepubertal children. The incidence of pediatric thyrotoxicosis is 0.1/100,000 in young children and 3/100,000 in adolescents [2, 4]. The majority of cases of pediatric thyrotoxicosis is secondary to Graves' disease, and patients typically present with weight loss, palpitations, tremors, tachycardia, and hypertension [5].

The incidence of thyroid storm is <10% of patients hospitalized for thyrotoxicosis, and it is usually associated with a precipitating event, such as infection, surgery, diabetic ketoacidosis, congestive heart failure, pulmonary embolism, toxemia of pregnancy, severe emotional stress, or discontinuation of antithyroid medications [6]. Thyroid storm has been reported after levothyroxine ingestion in a two-year-old patient [7]. Infection is the most common precipitating factor for thyroid storm. Symptoms of thyroid storm include tachycardia, moist skin, fever, agitation, weakness, systolic hypertension, tremor, delirium, palpitations, and altered mental status [8]. Table 2 shows Burch and Wartofsky's criteria for Thyroid Storm in adults, but there are no published criteria specific for pediatric patients. Our patient's scores are highlighted in bold.

Thyroid storm is a severe manifestation of thyrotoxicosis; the mortality rate ranges from 20 to 30% [6]. Treatment includes correcting the hyperthyroidism with antithyroid medication. Iodine can be used in addition to thioamides to decrease thyroid hormone synthesis. Restoring homoeostasis with IV hydration is essential, and beta-adrenergic blockers may be needed to control the cardiovascular manifestations. Glucocorticoids can be used to block conversion of thyroxine (T4) to triiodothyronine (T3) [2]. It is important to note that PTU is no longer recommended as a first-line agent in either children or adults for the management of Graves' disease due to safety concerns related to liver failure and death in children [9]. However, PTU may be preferable in patients with life-threatening thyrotoxicosis because of its additional inhibition of T4 to T3 and in pregnant women due to concerns of methimazole safety to the fetus [10].

## 3. Conclusions

We believe that this patient had underlying, undiagnosed Graves' disease. In addition, the tachypnea, wheezing, X-ray findings consistent with viral pneumonia and a positive RSV titer provide strong support for the conclusion that RSV infection triggered her thyroid storm. This case emphasizes the importance of doing a neck exam, including palpation of the thyroid gland, in patients who present with symptoms of respiratory distress and cardiovascular instability; thyrotoxicosis should be part of the differential diagnosis in these cases. A complete clinical history and physical exam are essential for early diagnosis and treatment of this potentially fatal disease.

## Conflict of Interests

The authors declare no conflict of interests.
